# The association between stress hyperglycemia ratio with mortality in critically ill patients with acute heart failure

**DOI:** 10.3389/fcvm.2024.1463861

**Published:** 2024-11-21

**Authors:** Tingai Ge, Jingjing Hu, Yidan Zhou

**Affiliations:** Department of Emergency Medicine, Hangzhou Third People’s Hospital, Hangzhou, China

**Keywords:** glucose, glycosylated hemoglobin, stress hyperglycemia, acute heart failure, critically ill, mortality

## Abstract

**Background:**

It's recognized that stress hyperglycemia ratio (SHR) is considered a significant indicator of poor prognosis in many diseases. However, its role in critically ill patients with acute heart failure (acute HF) remains underexplored.

**Methods:**

We conducted a retrospective cohort study on patients with acute HF included in the Medical Information Mart for Intensive Care IV (MIMIC-IV) version 2.2 database. A restricted cubic spline (RCS) regression analysis was used to explore the relationship between SHR and the risk of all-cause mortality in these patients. Subsequently, a Cox regression model was used to evaluate the relationship between SHR and mortality in acute HF patients.

**Results:**

A total of 1,644 acute HF patients were included in the study and divided into two groups: the low SHR group (SHR < 1.06, *N* = 823) and the high SHR group (SHR ≥ 1.06, *N* = 821). In our study, the 30-day, 90-day, 180-day, and 365-day mortality rates for acute HF were 7.0%, 12%, 15%, and 19%, respectively, with higher mortality rates observed in the high SHR group compared to the low SHR group. SHR levels showed a linear relationship with all-cause mortality. Furthermore, SHR as a continuous variable shows a significant positive correlation with 30-day (HR = 2.31, 95% CI: 1.58–3.39), 90-day (HR = 1.81, 95% CI: 1.31–2.52), 180-day (HR = 1.57, 95% CI: 1.16–2.12), and 365-day (HR = 1.41, 95% CI: 1.07–1.85) all-cause mortality. After categorization, high SHR remains associated with increased 30-day (HR = 2.4, 95% CI: 1.59–3.61), 90-day (HR = 1.76, 95% CI: 1.31–2.36), 180-day (HR = 1.51, 95% CI: 1.16–1.95), and 365-day (HR = 1.38, 95% CI: 1.09–1.73) all-cause mortality.

**Conclusion:**

Our findings indicate that high SHR is an independent predictor of poor short- and long-term prognosis in acute HF patients. Understanding the impact of SHR on mortality in acute HF is crucial as it can assist clinicians in identifying high-risk patients and adjusting treatment strategies accordingly.

## Introduction

Acute heart failure (acute HF) is a leading cause of hospitalization and mortality worldwide, imposing a significant burden on healthcare systems (1). In acute clinical settings, such as HF, stress hyperglycemia is commonly observed, characterized by transient elevations in blood glucose levels due to physiological stressors (2). It is typically reflective of the severity of the respective disease (3), induced through enhanced inflammation or activation of neurohormones (4). Research has shown that stress hyperglycemia is frequently observed in critically ill patients and is positively associated with mortality rates (4, 5). Moreover, it has been suggested as a potential indicator for predicting unfavorable outcomes in cerebrovascular disease (6–9), acute myocardial infarction (10–12), coronary artery disease (13), acute coronary syndrome (14), heart valvular disease (15), and sepsis (16, 17). In addition, recent studies have also highlighted the role of stress hyperglycemia in predicting adverse outcomes in patients with heart failure (18–22). Therefore, studying the impact of stress hyperglycemia on disease prognosis is crucial, as it can assist clinicians in identifying high-risk patients and adjusting their treatment strategies accordingly.

Previously, stress hyperglycemia was reflected by initial plasma glucose levels (23). However, plasma glucose levels are influenced by various factors (such as past glucose levels), which limits their ability to differentiate the state of stress hyperglycemia. To better assess the actual glycemic status of patients, the stress hyperglycemia ratio (SHR) has been proposed (24). The SHR is calculated as the ratio of an individual's acute glucose levels to their prior glucose levels, emphasizing the relative acute increase in glucose levels during stress responses or critical illness compared to their previous levels (24). Recent literature confirms that SHR reflects true stress hyperglycemia during hospitalization (11).

However, SHR in acute HF remains underexplored, particularly in critically ill patients, who have a worse prognosis. Therefore, we aim to elucidate the prognostic value of SHR by studying its impact on short-term and long-term mortality rates in this population.

## Materials and methods

### Data source

We conducted a retrospective observational study using the MIMIC-IV 2.2 database (25). It includes detailed medical records of patients hospitalized in the intensive care unit (ICU) at Beth Israel Deaconess Medical Center between 2008 and 2019. The institutional review boards of the Massachusetts Institute of Technology and Beth Israel Deaconess Medical Center approved the establishment of the database. Author Hu Jingjing has been authorized to use the MIMIC-IV database (ID: 52583254) following the completion of an online education program by the National Institutes of Health.

### Study population

**Figure 1:**
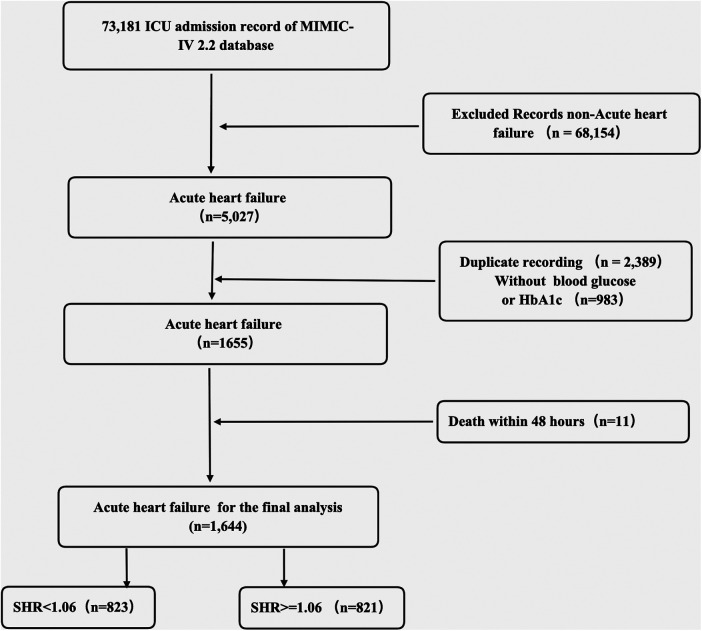
The flowchart of patients’ selection.

From the MIMIC-IV database, patients meeting inclusion criteria for acute HF were identified based on ICD codes (42821, 42831, 42841, I5021, I5031, and I50811). The inclusion criteria comprised adult patients (≥18 years old) admitted to the ICU. For patients with multiple admissions, only data from their initial admission were included. Exclusion criteria included lack of plasma glucose at admission or HbA1c data, discharge or death within 48 h, and insufficient follow-up information. Figure 1 depicts the patient selection process.

### Data extraction

A Structured Query Language (SQL) data extraction tool (pgAdmin 4) was utilized to retrieve the following data within the first 24 h of admission: (1) Demographic information including age, gender, and race; (2) Vital signs such as heart rate, respiratory rate (RR), systolic blood pressure (SBP), and diastolic blood pressure (DBP); (3) Pre-existing conditions including myocardial infarction (MI), congestive heart failure (CHF), chronic pulmonary disease, diabetes mellitus (DM), peripheral vascular disease, and cancer; (4) Laboratory tests conducted within the initial 24 h post-admission, encompassing hematocrit level, white blood cell count (WBC), platelet count, serum potassium level, serum sodium level, hemoglobin level, admission blood glucose, serum urea nitrogen level (BUN), serum creatinine level (SCR), anion gap, and other pertinent markers. The mean value was used for variables measured multiple times within the preceding 24 h; (5) Assessment of illness severity upon admission using the Simplified Acute Physiology Score II (SAPS II) and Sequential Organ Failure Assessment (SOFA) scores; (6) Administration of vasoactive medications, and mechanical ventilation (MV) during hospitalization; (7) Prognostic information includes ICU length of stay, total hospital length of stay, 30-day, 90-day, 180-day, and 365-day mortality.

### SHR

SHR is calculated as the first blood glucose at admission (mg/dl)/[28.7 × HbA1c (%) - 46.7] (24).

### Endpoints

The primary endpoint was 30-day all-cause mortality. Secondary endpoints included all-cause mortality rates within 90-day, 180-days, and 365-day.

### Statistical analysis

We conducted a restricted cubic spline (RCS) regression analysis to explore the relationship between SHR and the risk of all-cause mortality in acute HF patients. Subsequently, the cut-off value (1.06) at which the hazard ratio (HR) for 30-day mortality risk equals 1 were used to categorize patients into high SHR (SHR ≥ 1.06) and low SHR groups (SHR < 1.06). The fundamental clinical characteristics of patients were assessed in accordance with the low and high SHR groups. We used the Shapiro-Wilk test to assess normality. Categorical variables were presented as numbers and percentages (%), and comparisons were made using the chi-square test. For normally distributed continuous variables, data were expressed as mean ± standard deviation (SD) and compared using Student's *t*-test. For non-normally distributed continuous variables, data were presented as median [interquartile range (IQR)] and compared using the Mann-Whitney U test. Subsequently, a Cox regression model was used to evaluate the relationship between SHR and mortality in patients with acute HF. In this study, variables with a *P*-value < 0.1 in the univariate Cox regression analysis, as well as those known to be associated with acute HF prognosis, were included in the multivariate regression analysis. Model I adjusted for nothing; Model II adjusted for age, gender, and race; Model III adjusted for SAPS II score, SOFA score, MI, CHF, stroke, chronic pulmonary disease, DM, cancer, vasopressin use, and mechanical ventilation; and Model IV adjusted based on Models II and III. Finally, a subgroup analysis was conducted based on age, gender, myocardial infarction, diabetes, and cancer. Except for the stratification variable, the adjustment technique was the same as in Model IV. Data analyses were conducted using R statistical software version 4.3.0. A two-tailed *P*-value of less than 0.05 defined statistical significance.

## Results

Table 1 presents the baseline clinical characteristics of all participants. A total of 1,644 acute HF patients were included in the study and divided into two groups: the low SHR group (SHR < 1.06) and the high SHR group (SHR ≥ 1.06). In this study, the 30-day, 90-day, 180-day, and 365-day mortality rates for acute HF were 7.0%, 12%, 15%, and 19%, with mortality rates being higher in the high SHR group compared to the low SHR group. There were no significant differences between the two groups of patients in terms of age, gender, systolic blood pressure, platelet count, potassium level, SOFA score, SAPS II score, use of vasoactive drugs, mechanical ventilation, length of hospital or ICU stay, occurrence of CHF, peripheral vascular disease, cerebrovascular disease, chronic pulmonary disease, diabetes mellitus, and other diseases (all *P* > 0.05). Additionally, there were statistically significant differences between the two groups in terms of race, heart rate, respiratory rate, SpO2, WBC, hematocrit, hemoglobin, anion gap, BUN, creatinine, sodium level, and the occurrence rate of myocardial infarction (all *P* < 0.05).

**Table 1:** Baseline characteristics of participants.

Characteristic	Overall, *N* = 1,644	SHR < 1.06, *N* = 823	SHR ≥ 1.06, *N* = 821	*p*-value^2^
SHR	1.06 (0.87, 1.30)	0.87 (0.75, 0.97)	1.30 (1.17, 1.54)	<0.001
Age, year	70.44 (59.72, 79.56)	70.61 (59.86, 79.95)	70.26 (59.68, 79.44)	0.9
Male, *n* (%)	905 (55%)	459 (56%)	446 (54%)	0.6
Race				0.015
White	1,063 (65%)	537 (65%)	526 (64%)	
Black	226 (14%)	128 (16%)	98 (12%)	
Other	355 (22%)	158 (19%)	197 (24%)	
Heart rate, beats/min	85.00 (75.00, 98.00)	82.00 (74.00, 94.00)	88.00 (76.00, 102.00)	<0.001
SBP, mmHg	118.00 (102.00, 136.00)	117.00 (101.00, 135.00)	120.00 (103.00, 138.00)	0.089
DBP, mmHg	64.00 (53.00, 76.00)	62.00 (52.00, 74.00)	66.00 (55.00, 77.00)	<0.001
Respiratory rate, beats/min	19.00 (16.00, 23.00)	19.00 (15.00, 23.00)	20.00 (16.00, 24.00)	<0.001
Spo2, %	98.00 (95.00, 100.00)	98.00 (96.00, 100.00)	97.00 (95.00, 100.00)	<0.001
WBC (103/μl)	11.10 (8.20, 15.05)	10.40 (7.75, 14.35)	11.97 (8.79, 15.70)	<0.001
Platelets (103/μl)	197.50 (149.00, 261.50)	191.50 (148.00, 257.00)	203.25 (149.88, 268.13)	0.2
Hematocrit (%)	31.85 (27.85, 36.65)	31.25 (27.65, 36.10)	32.63 (28.20, 37.30)	0.003
Hemoglobin (g/dl)	10.40 (9.05, 12.10)	10.25 (9.00, 11.85)	10.60 (9.10, 12.30)	0.004
Aniongap (mmol/L)	14.50 (12.50, 17.00)	14.00 (12.00, 16.00)	15.50 (13.00, 18.00)	<0.001
Bun (mg/dl)	23.00 (16.50, 37.50)	22.00 (16.00, 35.00)	24.00 (17.50, 40.00)	<0.001
Creatinine (mg/dl)	1.15 (0.85, 1.75)	1.10 (0.85, 1.64)	1.20 (0.90, 1.90)	0.017
Sodium (mmol/L)	138.00 (135.50, 140.50)	138.50 (136.00, 140.50)	138.00 (135.00, 140.00)	<0.001
Potassium (mmol/L)	4.30 (3.95, 4.70)	4.30 (3.95, 4.63)	4.30 (3.95, 4.70)	0.3
Glucose (mmol/L)	134.25 (112.00, 172.00)	114.00 (100.00, 131.75)	162.00 (137.00, 209.00)	<0.001
HAb1c Comorbidities, *n* (%)	6.00 (5.60, 6.90)	6.20 (5.70, 7.30)	5.80 (5.40, 6.60)	<0.001
Myocardial infarction	532 (32%)	244 (30%)	288 (35%)	0.019
Congestive heart failure	1,122 (68%)	563 (68%)	559 (68%)	0.9
Peripheral vascular disease	211 (13%)	108 (13%)	103 (13%)	0.7
Cerebrovascular disease	195 (12%)	88 (11%)	107 (13%)	0.14
Chronic pulmonary disease	432 (26%)	219 (27%)	213 (26%)	0.8
Diabetes	720 (44%)	368 (45%)	352 (43%)	0.5
Cancer	153 (9.3%)	71 (8.6%)	82 (10%)	0.3
Therapy, *n* (%)				
Vasopresstion	147 (8.9%)	73 (8.9%)	74 (9.0%)	>0.9
Ventilation	709 (43%)	354 (43%)	355 (43%)	>0.9
Scores				
SOFA	4.50 (2.00, 7.00)	5.00 (2.00, 7.00)	4.00 (3.00, 7.00)	0.8
SAPSII Outcomes, days	35.00 (28.00, 44.00)	35.00 (28.00, 43.00)	36.00 (29.00, 45.00)	0.12
Los-icu	2.33 (1.27, 4.49)	2.25 (1.28, 4.12)	2.52 (1.26, 5.12)	0.082
Los-hospital	8.67 (5.14, 14.47)	8.79 (5.20, 13.84)	8.59 (5.04, 14.95)	0.8
30-day mortality, *n* (%)	115 (7.0%)	34 (4.1%)	81 (9.9%)	<0.001
90-day mortality, *n* (%)	194 (12%)	73 (8.9%)	121 (15%)	<0.001
180-day mortality, *n* (%)	242 (15%)	100 (12%)	142 (17%)	0.003
365-day mortality, *n* (%)	307 (19%)	134 (16%)	173 (21%)	0.013

### RCS analysis between SHR level and mortality

**Figure 2:**
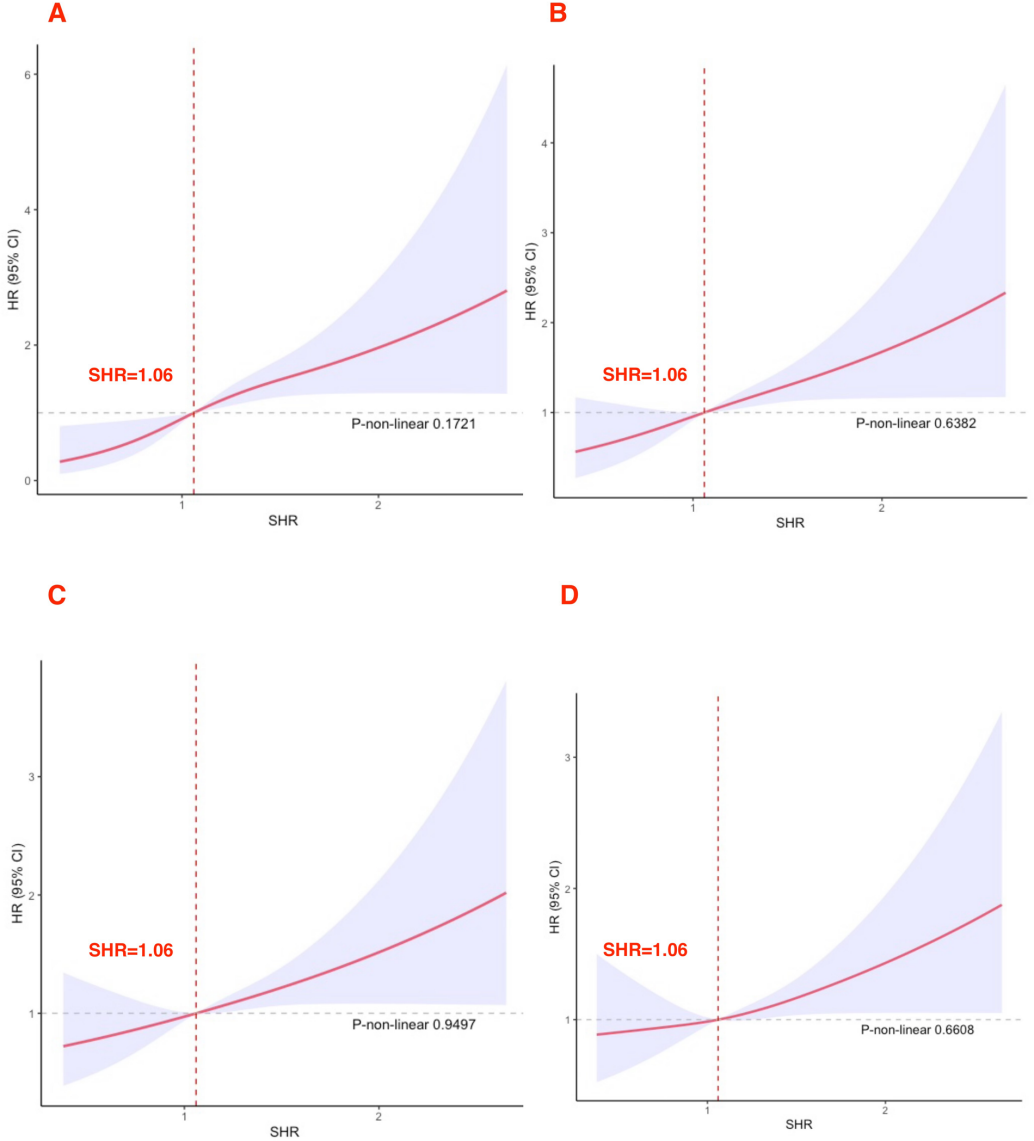
Cubic spline plot of the relation between high SHR and all-cause mortality **(A)** 30-day; **(B)** 90-day; **(C)** 180-day; **(D)** 365-day.

SHR levels showed a linear relationship with 30-day, 90-day, 180-day, and 365-day mortality rates (all *P* > 0.05). All RCS results are presented in Figure 2. When SHR exceeded 1.06, the risk of death in acute HF markedly increased.

### Relationship between SHR and all-cause mortality

**Figure 3:**
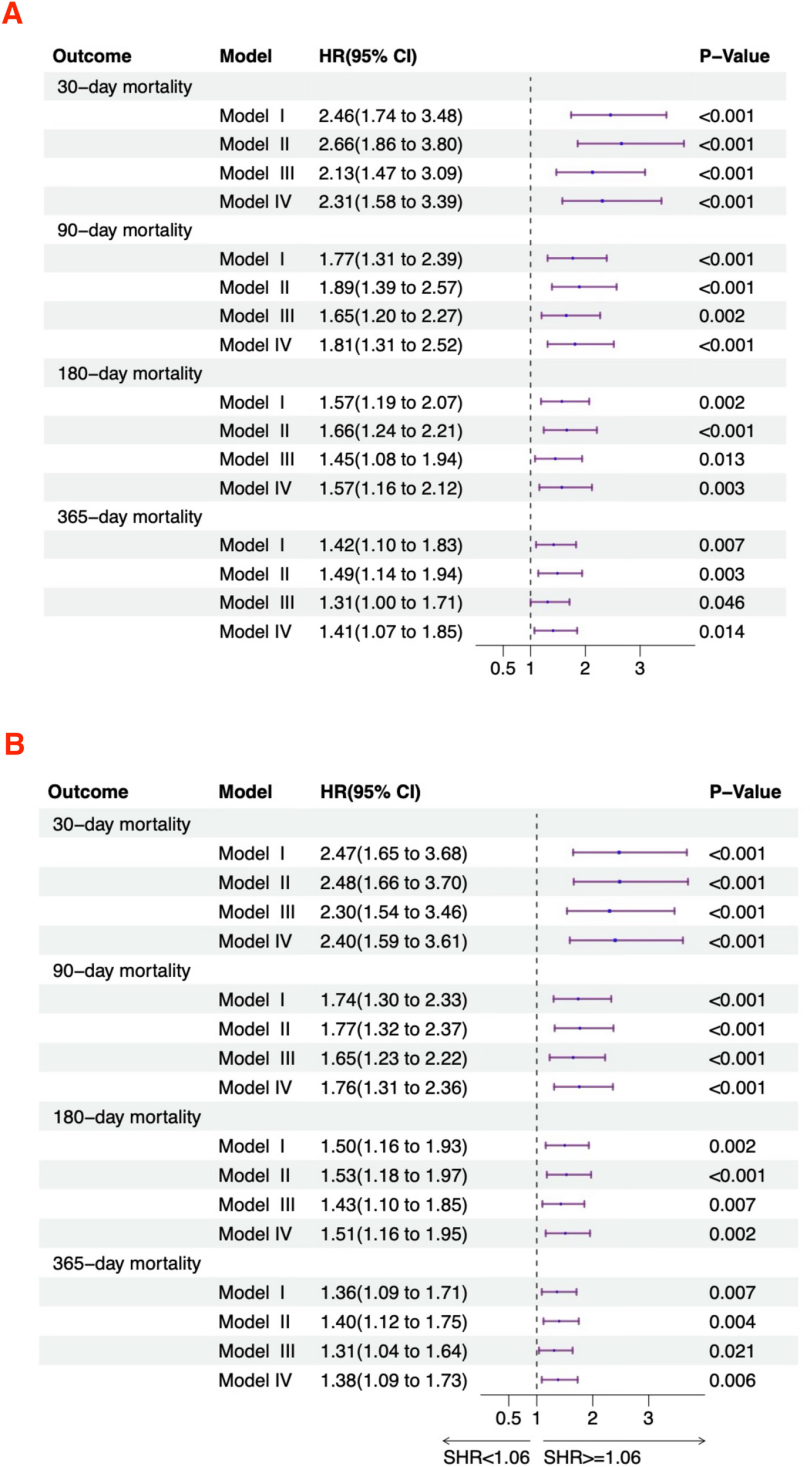
Hazard ratios for mortality based on SHR in acute HF patients. **(A)** Continuous variable; **(B)** Categorical variable.

Figure 3 displays the relationship between SHR and the risk of mortality in acute HF patients. In the unadjusted model (Model I), SHR as a continuous variable showed a significant positive correlation with 30-day (HR = 2.46, 95% CI: 1.74–3.48), 90-day (HR = 1.77, 95% CI: 1.31–2.39), 180-day (HR = 1.57, 95% CI: 1.19–2.07), and 365-day (HR = 1.42, 95% CI: 1.10–1.83) all-cause mortality rates. After stratification, high SHR remained associated with increased 30-day (HR = 2.47, 95% CI: 1.65–3.68), 90-day (HR = 1.74, 95% CI: 1.30–2.33), 180-day (HR = 1.50, 95% CI: 1.16–1.93), and 365-day (HR = 1.36, 95% CI: 1.09–1.71) all-cause mortality. In Model II, SHR as a continuous variable showed a significant positive correlation with 30-day (HR = 2.66, 95% CI: 1.86–3.80), 90-day (HR = 1.89, 95% CI: 1.39–2.57), 180-day (HR = 1.66, 95% CI: 1.24–2.21), and 365-day (HR = 1.49, 95% CI: 1.14–1.94) all-cause mortality. After categorization, high SHR remained associated with increased 30-day (HR = 2.48, 95% CI: 1.66–3.70), 90-day (HR = 1.77, 95% CI: 1.32–2.37), 180-day (HR = 1.53, 95% CI: 1.18–1.97), and 365-day (HR = 1.40, 95% CI: 1.12–1.75) all-cause mortality. In Model III, SHR as a continuous variable showed a significant positive correlation with 30-day (HR = 2.13, 95% CI: 1.47–3.09), 90-day (HR = 1.65, 95% CI: 1.20–2.27), 180-day (HR = 1.45, 95% CI: 1.08–1.94), and 365-day (HR = 1.31, 95% CI: 1.00–1.71) all-cause mortality. After categorization, high SHR was still linked to increased 30-day (HR = 2.30, 95% CI: 1.54–3.46), 90-day (HR = 1.65, 95% CI: 1.23–2.22), 180-day (HR = 1.43, 95% CI: 1.10–1.85), and 365-day (HR = 1.31, 95% CI: 1.04–1.64) all-cause mortality. In Model IV, SHR as a continuous variable showed a significant positive correlation with 30-day (HR = 2.31, 95% CI: 1.58–3.39), 90-day (HR = 1.81, 95% CI: 1.31–2.52), 180-day (HR = 1.57, 95% CI: 1.16–2.12), and 365-day (HR = 1.41, 95% CI: 1.07–1.85) all-cause mortality. After categorization, high SHR remained associated with increased 30-day (HR = 2.40, 95% CI: 1.59–3.61), 90-day (HR = 1.76, 95% CI: 1.31–2.36), 180-day (HR = 1.51, 95% CI: 1.16–1.95), and 365-day (HR = 1.38, 95% CI: 1.09–1.73) all-cause mortality.

### Subgroup analyses

**Figure 4:**
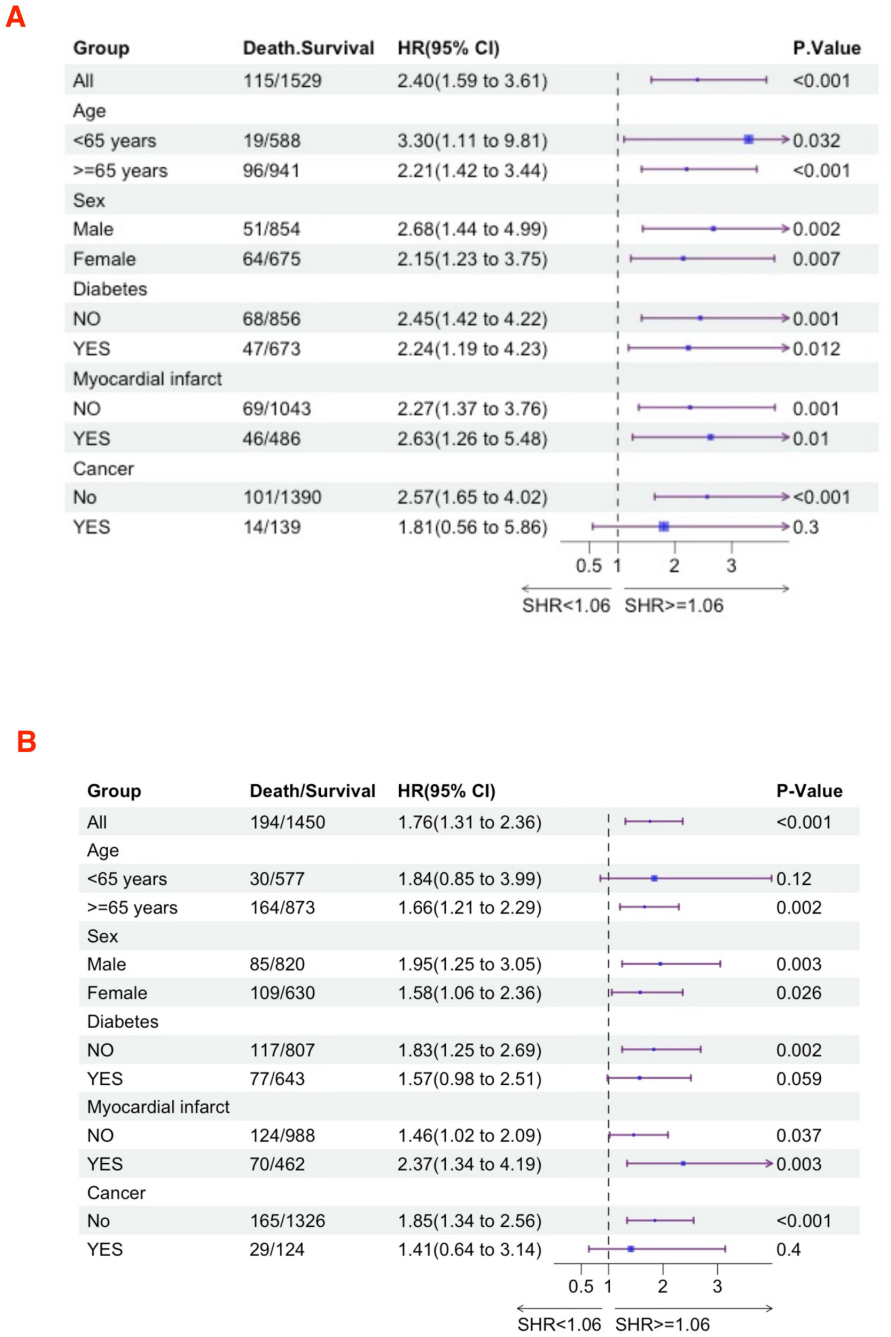
Subgroup analyses were performed to evaluate the association between high SHR and all-cause mortality **(A)** 30-day; **(B)** 90-day.

**Figure 5:**
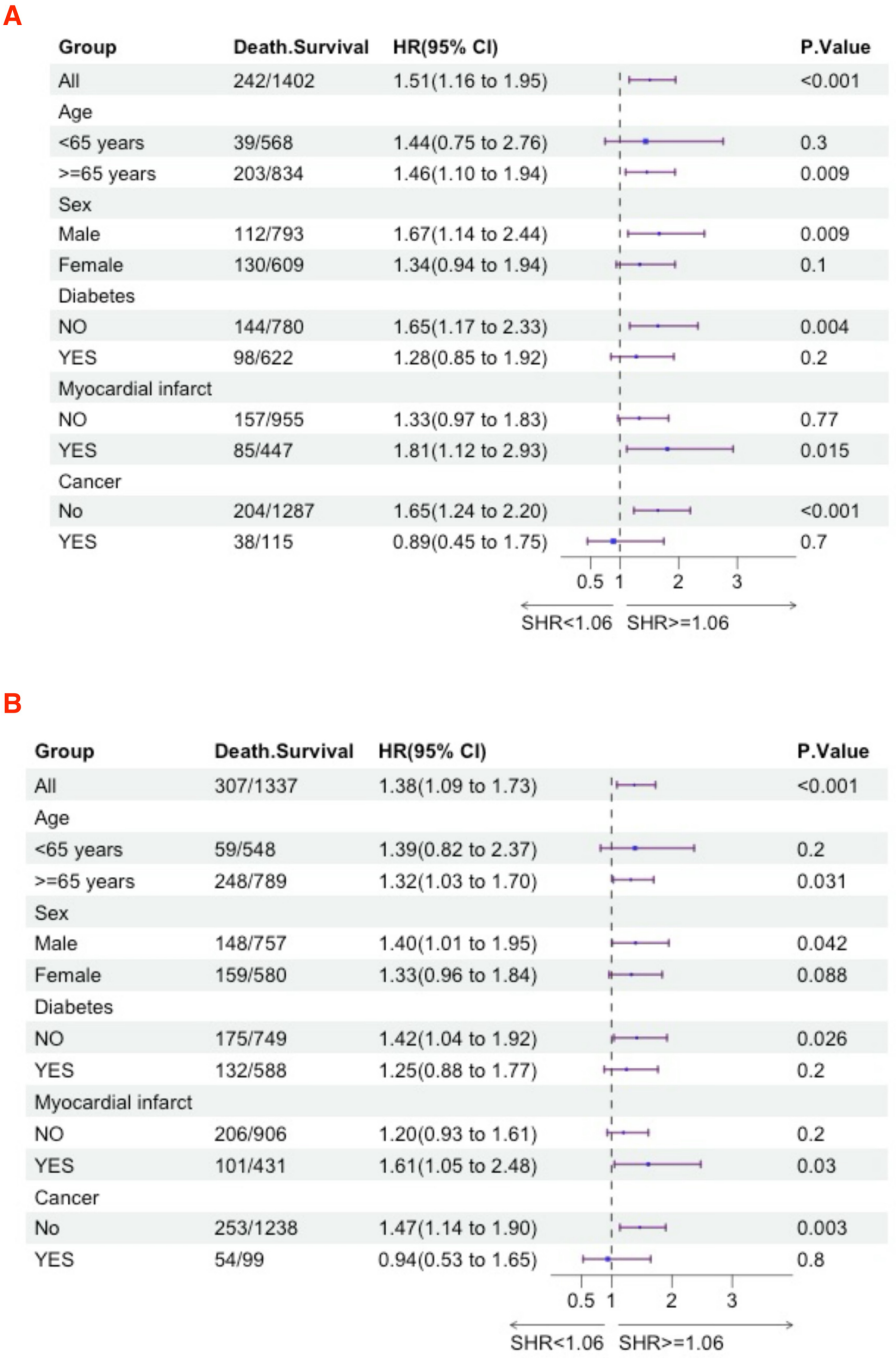
Subgroup analyses were performed to evaluate the association between high SHR and all-cause mortality **(A)** 180-day; **(B)** 365-day.

We further utilized subgroup analysis to evaluate the relationship between high SHR and all-cause mortality rates (Figures 4, 5). In the tumor subgroup, we did not observe an association between high SHR and mortality. However, high SHR was significantly correlated with the 30-day mortality in the remaining subgroups. For the 90-day mortality, we found that high SHR was associated with increased mortality in all subgroups except for those aged under 65 years with acute HF. Furthermore, we observed consistent results regarding SHR and mortality at 180 days and 365 days. Specifically, higher SHR was associated with mortality at both 180 days and 365 days in males, over 65 years of age, without diabetes, myocardial infarction, and in the non-tumor subgroup. In contrast, we found no association between high SHR and mortality at 180 days and 365 days in aged under 65 years, females, diabetes, or those without a history of myocardial infarction subgroup.

## Discussion

In this study, we explored the relationship between SHR and prognosis in critically ill acute HF patients, finding that high SHR is associated with short-term and long-term all-cause mortality rates. We identified a linear relationship between SHR in critically ill acute HF patients and all-cause mortality rates. When SHR exceeded 1.06, there was a significant increase in the risk of mortality. In this study, acute HF patients with serum SHR ≥ 1.06 compared to those with SHR < 1.06 had a 2.4-fold higher risk of 30-day all-cause mortality, a 1.76-fold higher risk of 90-day all-cause mortality, a 1.51-fold higher risk of 180-day all-cause mortality, and a 1.38-fold higher risk of 365-day all-cause mortality, respectively. Our study results suggest that SHR holds promise as a tool for assessing adverse outcomes in acute HF patients.

Previous studies have indicated that SHR is a predictor of poor prognosis in HF patients. Zhou et al. conducted a study on 2,875 patients with HF and DM. They found that, compared to the lower SHR group, the adjusted odds ratio (OR) for composite cardiac events in the high SHR group was 1.89 (95% CI 1.26–2.87; *P* = 0.002) (20). Similarly, Mohammed et al. conducted a study on 400 patients with HF with preserved ejection fraction, followed for an average of 41 months. They found that elevated SHR was independently associated with an increased risk of all-cause composite events, cardiovascular death, and heart failure rehospitalization compared to patients with lower SHR (adjusted HR: 2.34, 95% CI 1.49–3.67; *p* < 0.001) (21). Khan also found similar conclusions. Furthermore, they demonstrated that patients over 65 years old had a 1.8 times higher likelihood of experiencing major adverse cardiovascular events compared to younger patients (95% CI: 1.1–2.9; *P* < 0.05), emphasizing age as a critical factor in risk stratification (22). Our study supports this conclusion as well. Apart from 30-day mortality, other outcomes indicate that high SHR is associated with a significantly higher risk of death in the population over 65 years old compared to younger individuals. Zhou et al. conducted a median 3.24-year follow-up study on 1,904 patients with acute HF. They found that acute HF patients in the highest SHR quintile (SHR > 1.14) had significantly higher risks of all-cause mortality (HR 2.76, 95% CI 1.63–4.68), cardiovascular (CV) mortality (HR 2.81, 95% CI 1.66–4.75), and HF rehospitalization (HR 1.54, 95% CI 1.03–2.32) compared to those in the lowest SHR quintile (0.64 < SHR ≤ 0.77). Additionally, they observed a U-shaped relationship between SHR and all-cause mortality (18). In our study, we found a linear relationship between SHR and both short-term and long-term mortality. This difference may be attributed to variations in the study population and follow-up duration. Our study exclusively included acute HF patients admitted to the ICU, who had higher comorbidities compared to those in previous studies. Therefore, they are at a higher risk of mortality. Our study shows that SHR has predictive value not only for long-term mortality but also for higher short-term mortality risk. In addition, contrary to our findings, Carrera et al. discovered a negative correlation between SHR and mortality. Compared to the first quintile, the HR for the second and third quintiles were 0.76 (95% CI: 0.58–0.99; *P* = 0.046) and 0.68 (95% CI: 0.52–0.89; *P* = 0.005), respectively (19). They found that patients with low SHR had hypoglycemia, which was negatively correlated with HF mortality rates.

The precise mechanism linking stress hyperglycemia in acute heart failure patients to adverse outcomes is not fully understood at present. First, stress hyperglycemia typically results from complex interactions of hormonal regulation (such as catecholamines, glucocorticoids, and cytokines) during stress or illness phases (26). It may be induced through activation of the sympathetic nervous system and enhanced activity of the hypothalamic-pituitar*y* axis (27). Additionally, increased sympathetic nervous activity can promote the release of glucagon, which in turn stimulates glycogen breakdown in muscles and the liver. This leads to an increase in glucose entering circulation, ultimately resulting in elevated blood glucose levels (28). The mechanisms of heart failure involve various factors, including systemic inflammation, endothelial dysfunction, myocardial fibrosis, and diastolic dysfunction (29). Studies indicate that stress hyperglycemia can also lead to endothelial dysfunction, oxidative stress, and inflammation (30–32), as well as activate coagulation (33, 34). These changes can cause diminished cardiac function (35), facilitate fluid retention, and worsen HF symptoms. Secondly, stress hyperglycemia often indicates relative insulin deficiency, leading to increased fat breakdown and elevated circulating free fatty acid levels (27). The increase in circulating free fatty acids can be toxic to the myocardium, exacerbating myocardial cell damage, calcium overload, and arrhythmias. Insulin deficiency also reduces myocardial anaerobic glucose utilization capacity (36). Under these conditions, myocardial damage is further exacerbated. Finally, stress hyperglycemia can increase the risk of infection and exacerbate other comorbidities, potentially leading to non-cardiovascular-related mortality (37). Therefore, stress hyperglycemia occurs due to inflammation and neuroendocrine disturbances in HF. This, in turn, worsens the prognosis of heart failure patients by exacerbating oxidative stress, inflammatory states, and endothelial dysfunction. However, the mechanisms underlying the relationship between stress hyperglycemia and outcomes in HF patients require additional research.

We investigated the prognostic correlation between SHR and acute HF patients in the MIMIC database. However, our study has some limitations. Firstly, this is a retrospective study, and despite including numerous variables, there may still be potential confounding factors that were not accounted for. Additionally, due to limitations inherent in database studies, many factors related to HF (such as NT-proBNP, cardiac enzyme profiles, troponin, and echocardiography) were not included in this study. Finally, despite the large sample size in our study, the findings are only applicable to a specific subset of critically ill AHF patients in the United States. Further research is needed to determine its applicability to other populations. Furthermore, further studies should be performed to explore the relationship between SHR and the prognosis of acute HF patients.

## Conclusion

Our findings indicate that high SHR is an independent predictor of poor short- and long-term prognosis in acute HF patients. Understanding the impact of SHR on mortality in acute HF is crucial as it can assist clinicians in identifying high-risk patients and adjusting treatment strategies accordingly.

## Data Availability

The original contributions presented in the study are included in the article/Supplementary Material, further inquiries can be directed to the corresponding authors.
